# World first pig‐to‐human cardiac xenotransplantation

**DOI:** 10.1111/xen.12733

**Published:** 2022-02-16

**Authors:** Wayne J. Hawthorne

**Affiliations:** ^1^ The Centre for Transplant and Renal Research Westmead Institute for Medical Research Westmead New South Wales Australia; ^2^ Department of Surgery School of Medical Sciences University of Sydney Westmead Hospital Westmead New South Wales Australia

1

On January 7, 2022, in Baltimore, MD, USA, the XenoHeart team at the University of Maryland School of Medicine (UMSOM) led by Dr. Muhammad Mohiuddin (President Elect International Xenotransplantation Association [IXA]) and Dr. Bartley Griffith performed the world's first successful pig‐to‐human cardiac xenotransplant. The recipient Mr. Bennett, a 57‐year‐old male on veno‐arterial extracorporeal membrane oxygenation (VA‐ECMO) with end‐stage nonischemic cardiomyopathy (NICM), received a pig heart with 10 gene modifications. Permission for this procedure was granted under an expanded access authorization by the United Stated, Food and Drug Administration (FDA) (also known as “compassionate use”). The patient is now over a week post operatively from the transplant, he is off VA‐ECMO, extubated, and on no supportive inotropic agents with a normal cardiac index and normal biventricular function as demonstrated by echocardiography. This transplant comes from the culmination of countless years of dedication and work by members of the International Xenotransplantation Association (IXA). Many people have made significant contributions to the broader field of xenotransplantation, which includes research, and policy development with extensive collaboration between the IXA, The Transplantation Society, and the World Health Organization along with regulatory authorities of individual nations, such as the FDA. This breakthrough would not have been possible without their efforts, support, and guidance. We share this outstanding achievement with all of you, in the field of Xenotransplantation.

As a background to this achievement, it has taken decades of research that has led to this outcome, which started in 2005 with the cardiac xenotransplantation program at the National Institutes of Health using nonlife supporting model of cardiac xenotransplantation (i.e., the heterotopic abdominal model). At that time rejection‐free survival of a cardiac xenograft with three gene modifications in a pig was up to 945 days in a nonhuman‐primate (NHP) model and was achieved using costimulation blockade‐based immunosuppression. At the UMSOM in Baltimore, MD, transition from heterotopic to life supporting orthotopic cardiac xenotransplantation occurred, and consistent long‐term survival of genetically engineered xenografts was achieved.

For this transplant to occur, the Maryland team applied to the FDA for expanded access or compassionate use authorization (i.e., treatment designed for patients with immediate life‐threatening conditions to obtain access to investigational products outside of an FDA‐approved clinical trial when no comparable or alternative therapeutic treatment exists to treat the patient's illness). After a rigorous review of preclinical data from NHP's provided by the UMSOM team to the FDA, approval for the first pig‐to‐human heart transplant was granted. The recipient Mr. Bennett was deemed to not be a suitable candidate for heart allotransplantation/ventricular assist device by UMSOM /UMMC multidisciplinary selection committee, other regional centers, and an external clinical advisory board but agreed that the risk of death was likely 100%. Mr. Bennett was offered a pig xenograft and he consented to the experimental procedure. Internal and external psychiatric evaluations were performed. Ethical and Institutional Review Board (IRB) approvals were obtained. The transplant was successfully performed without any issues, and the patient is making steady recovery.

The IXA Council and entire field congratulate the Maryland team for their achievement and acknowledge many teams involved and authorities including the FDA that gave permission for this to occur. This is a major step forward for the field and a wonderful achievement for a very sick patient.


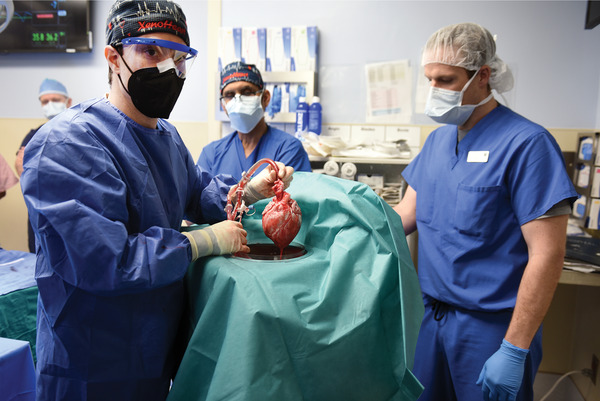


## CONFLICT OF INTEREST

The author declares that there is no conflict of interest that could be perceived as prejudicing the impartiality of the research reported.

